# A Comparison between the Methodology of the Mainstream in (Neuro-)Psychology, Holzkamp’s and Vygotsky’s Approach

**DOI:** 10.1007/s12124-024-09880-6

**Published:** 2024-12-28

**Authors:** Leonard Nigrini, Federica Amici, Miquel Llorente

**Affiliations:** 1 Comparative Minds Research Group, Departament de Psicologia, Universitat de Girona, Plaça Sant Domènech 9, Girona, 17004 Catalonia Spain; 2 Human Biology and Primate Cognition, Institute of Biology, Faculty of Life Science, Leipzig University, Leipzig, Germany; 3 Department of Comparative Cultural Psychology, Max Planck Institute for Evolutionary Anthropology, Leipzig, Germany

**Keywords:** Vygotsky, Holzkamp, Neuropsychological methodology, Critical psychology, Dialectics

## Abstract

The present paper treats the issue of methodological assumptions in mainstream neuropsychology and, as counter-concepts, in Vygotsky’s approach and Holzkamp’s critical psychology. The analysis identifies four main assumptions concerning the methodology of mainstream neuropsychology, which are contrasted with the positions of other approaches. The methodologies of the mainstream neuropsychology vs. Holzkamp’s and Vygotsky’s approach assume: (1) mechanistic vs. dialectical materialism; (2) formal vs. dialectical logic; (3) decomposition into elements vs. units; (4) reductionism of psychic processes to the brain vs. activity as a unity of environmental and organism-pole. Despite the vast coincidence in their main assumptions, we also discuss nuances of difference between Holzkamp’s and Vygotsky’s approaches. The former, possibly due to its reference to cultural-historical activity theory (CHAT) and its theoretical neglection of the organism-pole of psychic functions, falls short of structural considerations in its accounts on phylogenetic emergence. On the other hand, Vygotsky’s neuropsychology does not fully explore the phylogenetic emergence of basic units of functional psychic organisation. This might be due to certain implications of Vygotsky’s initial accounts, which seem to highlight cultural development to the detriment of phylogenetic one.

## Introduction

In 1973 the soviet neuropsychologist A.R. Luria wrote:

“[W]e can only predict that in the next fifty years our views on the structure of mental processes will differ substantially from those which we hold today; neuropsychology will deserve much of the credit for this revision and deepening of our knowledge of the internal structure of mental processes” (p. 343).

At the time of commencing to write this paper, it is the year 2023, marking exactly fifty years since Luria predicted significant advances in the field of neuropsychology. Thus, today one should be able to see the fruits of this scientific progression. Indeed, in part this promise has been kept. Many brilliant scholars have built on the legacy of Luria and Vygotsky in neuropsychology. Tatjana Akhutina, Marta Shuare, Aaro Toomela and Anne Christensen are maybe among the most famous examples for such genius researchers (cf. García, 2022; cf. Herreras, 2006; Valsiner & Cornejo, 2019, p. vi). In spite of all the well deserved credit being given to these oustanding scientists, the global picture being painted for the state of neuropsychology is far less encouraging. With a replication rate of only 60% (Open Science Collaboration, 2015) neuropsychology neither does appear to live up to its proclaimed role as leading science for the study of mind (Maiers, 2008) nor does it seem to present an exception from the recent general methodological crisis in psychology.

The mainstream of contemporary neuropsychology has evolved in ways distinct from what Luria had envisaged as the path to significant advancement. This paper aims to identify methodological assumptions of the modern mainstream neuropsychology and to contrast them with the position of Vygotsky’s (Luria’s) and Holzkamp’s approaches as proponents of an alternative methodology (cf. van Ijzendoorn et al., 1984; cf. Kölbl, 2023). Despite sharing similar social and epistemological objectives, the cohesion between the traditions of Vygotsky and Holzkamp is relatively sparse. We further aim to discard notions of inconsumerability of these approaches and demonstrate their potential to be integrated into a framework for the investigation of functional psychic organisation.

## Methodological Foundation of the (Neuro-) Psychological Mainstream

### Introductory Remarks

Modern neuropsychology is not a homogenous field. It lacks a universally accepted methodological manifesto that underlies its research and theory. Nevertheless, it is possible to identify a general tendency towards certain (non-expressed) methodological assumptions. Mainstream psychology and neuropsychology are, according to Toomela (2007, p. 10), “a direct continuation of pre-WWII North American psychology”, which is concerned rather with the “accumulation of facts[, ] than with elaborations of the theoretical meanings of these facts”. Other definitions refer to it as “nomothetic science”, due to its inclination to formulate universal theoretical principles (Holzkamp, 1972, p. 52), or as “bourgeois” psychology, due to its social embedding (cf. Jäger & Staeuble, 1978, p. 321). However, these definitions only capture certain aspects of its essence. The subsequent analysis will unveil further features that could as well be used in the definition of mainstream neuropsychology. Since there are no explicit statements or declarations that definitely establish the subsequently described principles as assumptions of the methodology of the neuropsychological mainstream, the analysis itself takes the form of an assumption (about the assumptions of the methodology of the mainstream).

### First Assumption: Mechanistic Materialism

Mainstream neuropsychology mostly relies on brain measurements to derive explanations about human psychological processes. A series of interviews conducted by Wengemuth (2022) revealed that neuroscientists virtually unanimously consider psychic processes to have a material substrate. Often researchers expressed their belief that human psychological processes and biological processes are identical or could be treated as such. Several researchers stated either that they “assume[…] the methodological identity of neuronal and psychic processes” or think that “mental processes can be reduced to our [human] biology” (Wengemuth, 2022, p. 99 f.). Statements like these are interpreted as indicating the assumption of materialism (Wengemuth, 2022, p. 190). There are various publications in which neuropsychologists stress their position in line with monistic materialism and in contra of dualist positions that understand psyche and body as different substances (Wengemuth, 2022, p. 9). Empirical studies like Libet’s (1985) experiment on the neuronal predecessors of voluntary movements, which are supposed to argue against dualist views on the psycho-physical problem, grant a deeper understanding of the type of materialism underlying mainstream neuropsychology. In this experiment, participants were asked to perform voluntary actions (such as moving their hand), while indicating the point on which the intention to do so became conscious to them. By using electroencephalography (EEG), Libet (1985) identified a so called ‘readiness potential’, which preceded the point at which participants indicated a conscious decision.

Based on Libet’s (1985) experiment and similar studies, Goschke (2004, p. 192) concludes that “the effect of intentions consists in the variation of attractor states that are approached by sensory and motor systems in consequence to a specific stimulus”. In other words: psychic processes, which are (supposedly) synonymous to neuronal processes, are ultimately understood as functions of external causes. This highlights that the materialism of mainstream neuropsychology can be characterized as a mechanistic materialism. In mechanism, “any change of movement is carried into a thing from outside and does not spring from its own inner constitution” (Conze, 2016, p. 131). This is supported by the almost universal practice of mainstream neuropsychological investigations to use experimental designs in which an external stimulus, a so-called independent variable, is supposed to induce changes in a dependent variable representing some kind of psychological processes. The assumption of a mechanistic materialism is not only very common in the neuropsychological mainstream, but it is also directly reflected in the concrete methodical principles of this field.

### Second Assumption: Formal Logic

Formal logic and the assumption of mechanistic materialism are grounded in a shared approach to the principle of contradiction (PC). The principle of contradiction can essentially be expressed by the idea that “the same attribute cannot at the same time belong and not belong to the same subject in the same respect” (Heine, 2016, p. XXX). For the validity of the PC the principle of identity (PI) is required. The essence of the PI is that “everything is equal to itself” (Engels, 1975, p. 484). Because the PC assumes identity, “the mere fact of change or of becoming is advanced […] as a decisive case against the objective validity of the principle of contradiction” (Conze, 2016, p. 62). Formal logic therefore excludes becoming, since change itself is contradictory, and contradiction is seen as equal to not-being. In formal logic, the being of things equals their remaining. Since proper becoming implies contradictions, formal logic is unable to deal with it. Thus comes the pairing of formal logic with mechanism. Since change in mechanism is always caused by an external factor and is thus external to the things, there is no contradiction. This allows to perceive the things as identical beings —the PC is not violated.

Formal logic can be considered a methodological fundament of mainstream neuropsychology. Wengemuth (2022, p. 31, p. 178) describes how the main or sole reference to scientific methodology that neuropsychologists reported during her interviews was Karl Popper’s neo-positivism. One of the interviewed neuroscientists stated: “in my impression the only thing [regarding scientific methodology] covered within the psychology degree is a simple version of Popper[‘s theory]” (Wengemuth, 2022, p. 167). Popper’s neo-positivism relates to formal logic as it determines that valid scientific conclusions must be derived from the application of modus tollens (falsificationism), a principle of logical inference, which infers the absence of the premises from the absence of their consequence.

Popper’s neo-positivism, at least in the oversimplified form to which Wengemuth’s (2022) interview partners referred, is a derivate of formal logic (cf. M. Jäger & Leiser, 1979, p. 40 f.), as here true statements are to be deducted via modus tollens. This supposes a logic of the things as identical beings —formal logic. The principle of falsificationism implies an approach to the validity of the PC in which contradictions equal not-being; therefor, formal logic serves as the substructure of Popper’s theory.

### Third Assumption: The Analytic Principle of the (Neuro-)Psychological Mainstream

Another very common principle in mainstream neuropsychology is the assumption that complex phenomena can analytically be taken apart and reduced to the sum of the characteristics of their parts (atomism), so that social and psychological phenomena can be explained on the basis of their underlying components (cf. Wengemuth, 2022, p. 176). The popularity of the analytic approach in the field of neuropsychology is demonstrated by the fact, that “the neurosciences were very successful in the decomposition of the whole into its components. For example, they are able to tell off which areas, cell layers, neurons, synapses, etc. the brain is composed” (Wengemuth, 2022, p. 39). At the same time, they still struggle to explain how these components contribute to the emergence of the whole (e.g. psychic processes or consciousness; Wengemuth, 2022, p. 101). Another example of the analytic principle is the assumption of the modularity within the brain. It is often assumed that specific areas within the brain serve distinct psychological functions (e.g. Karnath, 2003, p. 2). More current accounts mostly abandon this notion of modularity in favour of so-called neuronal networks. However, it is questionable to what extend this is a completely different understanding or a mere development of modularity, since neuronal structures are still identified with a certain part of a psychological function (instead of the function as such). As shown in the subsequent elaborations, such a mere augmentation of the degree of detail does not lead to a better systemic understanding of a phenomenon in question, if its parts are analysed as elements rather than as units. The analytic approach in the neuropsychological mainstream can help to understand another very recurrent tendency in the field: methodical specification. If the understanding of a complex whole equals the understanding of its components, then decomposing its components to a gradually more basic level must be seen as the obvious way to improve the understanding of the thing as a whole. Thus, in mainstream neuropsychology, methodical advance and specification are understood as the apparent way to fill the gaps within its understanding. There seems to be a widespread belief among neuropsychologists that methodical specification and the appearance of advanced methods are able to solve the most basic problems of their science, including those of categorical nature (pertaining to the meanings of the categories that neuropsychology investigates; cf. Holzkamp, 1972a; Wengemuth, 2022, p. 133).

The current trend towards analytical decomposition into elements relates to the assumption of mechanistic logic, which considers all phenomena as effects of different external phenomena, forming an infinite chain of causation. Since causation is presumed to originate from external sources, the relation between the parts and the whole supposes a certain form: the parts cannot be units of the whole and cannot be understood from the perspective of the whole, the relation of the parts to the whole must be reductive, and the whole is seen as equivalent to the sum of its parts, as inner characteristics could not be causes (Engels, 1975, p. 57, p. 315). Atomism, or analytical decomposition, is therefore the obvious consequence of the assumption of a mechanistic materialism.

### Fourth Assumption: Reductionism

The tendency of reductionism in mainstream neuropsychology relates in a broad sense to the previously discussed assumptions of the analytic principle and atomism. Reductionism refers to the understanding that scientific progress is mediated by the discovery of reductive relations: complex phenomena can be disassembled into their components, whose characteristics can explain the phenomenon as a whole (cf. Kötter, 1992, p. 372). Although, the notion of reductionism resembles accounts on the analytic principle, here we refer to reductionism in terms of the reduction of psychic processes to neuronal processes or even exclusively central nervous processes. This assumption is rather common among neuropsychologists (Wengemuth, 2022). Wengemuth (cf. 2022, p. 101, p. 107, p. 137) reports on several instances the conviction that psychic and neuronal processes are to be understood as synonymous. According to this conception, it can be inferred that within the mainstream of neuropsychology a form of naturalistic conviction prevails. Naturalism describes the understanding that everything within the world can be explained with the same natural principles (physics, biology, chemistry; cf. Gawlick, 1984, p. 517 f.). Several studies have attempted to locate specific psychological functions (e.g., executive function) into specific areas of the brain, providing support to the alleged reduction of psychic processes to the (central) nervous system (e.g. Banich et al., 2000; Potenza et al., 2003). The naturalism/reductionism of mainstream neuropsychology is especially evident in these studies, which treat complex psychic functions as properties of the organic matter of the human nervous system.

## The Positions of Holzkamp’s Critical Psychology and Vygotsky’s Approach towards the Methodology of the (Neuro-)Psychological Mainstream

### Background

Other than mainstream neuropsychology, Holzkamp and Vygotsky explicitly disclose their methodological assumptions. In Holzkamp’s opinion, there are four different planes according to which scientific insight is positioned and which describe its methodological and epistemological basis. The most general plane according to Holzkamp is the ‘philosophical plane’. He states that on this plane ‘his’ critical psychology refers to dialectical materialism as its base. Similarly, Vygotsky “accept[s] the realistic, objective, i.e., the materialistic viewpoint in epistemology and the dialectical viewpoint in logic” (Vygotsky, 1997a, p. 225). Subordinate to the philosophical plane is the ‘socio-theoretical plane’. Both Holzkamp and Vygotsky associate their approaches on this plane with historical materialism, which refers to Marx’ and Engels’ analysis of the development and characteristics of the bourgeois society (cf. del Rio & Álvarez, 2016; Holzkamp, 1985, p. 27; Tolman, 1994, p. 35; Vygotsky, 1986, p. 94 f.). Further, Holzkamp mentions the ‘categorical plane’ which refers to the basic terms of an empirical science to describe its subject. He states that these categories are related to the assumptions made by science about the philosophical and socio-theoretical plane, but not unequivocally determined by it. According to Holzkamp, critical psychology as a paradigm is principally occupied with the revelation of psychological categories. However, there is a fourth plane, the ‘plane of singular theories’, which relates to concrete theories about empirical issues. The theories on this plane relate to the categorical plane insofar as they are formulated using its terms (Holzkamp, 1985, p. 27 f.). Other than Holzkamp’s critical psychology, Vygotsky’s approach refers to both third and fourth plane. Though he sees the “[t]he psychological language of contemporaneity is [as] terminologically insufficient” (Vygotsky, 1997b, p. 281) and calls for the clarification of categorical meanings, a broad extent of Vygotsky’s works is also located on the plane of ‘singular theories’.

### Position of Holzkamp’s and Vygotsky’s Approach towards the First Assumption of (Neuro-) Psychological Mainstream

**The Contradictory Character of Mechanistic Materialism.** The emergence of modern psychology coincided with a period of important changes in society and production, namely the use of automated machinery which enabled great advances in the appropriation and domination of the natural world. According to Conze (2016) this social phenomenon was accompanied by a change of attitude in science: “The question about the being of things has been abandoned. It is sufficient that they obey” (p. 203). Simultaneously, the dissolution of feudal hierarchy lead to an increasing abandonment of the idea that humans were determined by their social position. Instead, a new notion of understanding humans as yet another part of nature became increasingly popular (Jäger & Staeuble, 1978, p. 322). Given this naturalistic understanding of humans and the general tendency to aspire for domination of the world rather than its understanding, a demand for an approach that pretends to offer control over human psyche and activity was present. Mechanistic materialism offers to explain everything by something external, thus providing the means to meet this demand of potential control.

It has been mentioned earlier, that

“[a]s far as the relation of inner and outer is concerned, everything inside appears as something outside to mechanics. The world is to be taken apart into units in such a way that it is always something outer that carries determinations into things; each determination as such is to be understood as external. (…) Consequently, a ghostly reality of points results, of empty transit point, which are filled by empty transit points and which receive their final fulfilment in God” (Conze, 2016, p. 288).

Mechanistic explanations imply a chain of causation - everything is to be explained by something else (Engels, 1975, p. 315). However, since the explaining phenomenon itself must be explained by another external thing, the chain of explanation is continued to infinity. Since circular proofs actually do not proof anything (Conze, 2016, p. 167), the chain of causation requires the assumption of something independently existing as the first cause of everything else. This first thing cannot be explained with the logic of mechanistic materialism, as it cannot be caused by something exterior to it. The first cause cannot be captured in materialist categories; it has to be something metaphysic, with an idealist notion (Conze, 2016, p. 288). Therefore, mechanistic materialism is based on a very contradictory fundament: it operates within the context of material phenomena and it uses empirical methods for the investigation of its subjects. However, its basis is idealist and that translates to the use of concepts and categories that have no material basis. In a way, it is remarkable how the neuropsychological mainstream “finds itself trapped in the same dualist way of thinking that it pretends to criticize” (Maiers, 2008, p. 59 ).

**Dialectical Materialism as the Basis of Holzkamp’s and Vygotsky’s Approach.** Both Holzkamp and Vygotsky base their approaches on dialectical materialism (Holzkamp, 1985, p. 27; Tolman, 1994, p. 35; Vygotsky, 1997a, p. 256). While in mechanistic theory the principle of contradiction is interpreted in the way that contradictions are the opposite of being —that they describe something impossible, a non-being, the dialectical approach considers contradictions to be real, and further to effectuate motion and development (Holz, 2005, p. VIII). Whenever contradictions occur, motion is set into place, so that contradictions indeed cannot be seen as something that ‘is’, but rather as motion, something becoming. The dialectical logic does not violate the principle of contradiction: everything that is- that remains- has no contradiction in it. However, when contradiction occurs, it sets things into motion, it terminates their remaining. In dialectical theory, change and motion are actually the standard while persisting is an exception, a special case of movement. Things are in constant change, since

“all things [are understood] as (historical) processes, that is, in their own movement and steady change and metamorphosis and presupposes the reality of movement and of contradiction in it; everything that exists, insofar as it is transitional, will contain contradictions, and has to be considered as transitional. The movement of a ‘thing’ has its origin in its very own constitution. The inner lawfulness of things is not only designed for self-preservation, but also for transformation of self into an other [sic], but which belongs to its self itself [sic]. Self-preservation is not denied, but dialectically understood” (Conze, 2016, p. 131).

Dialectics consider motion as the basic principle of things. The so called ‘own movement’ reflects their inner contradiction, whose opposite poles dissolve into a new unity, transforming the thing into something new. The dissolving of the inner contradiction into a new unity does not make it vanish, but it supersedes it in the new synthesis as a unit. In dialectics, change is not explained as the result of external factors but rather as the result of inner own movement. This does not cancel out the influence of environmental factors, but these are considered part of the internal contradiction setting things in motion, rather than external factors (Engels, 1975, p. 43).

“[O]ne notices a contradicting part in a state of affairs, then one will not trace it back to opposed external forces that external to it, even though they collide in it […]; rather, one will grasp it as an inner contradiction of inner properties and tendencies, that are proper to itself” (Conze, 2016, p. 288).

For mechanistic materialism, it is impossible to grasp how something new can emerge from change, whereas dialectics suppose the transition of quantitative into qualitative change. Here, change is an inherent part of all things, while mechanism considers things to normally be in rest and only due to an external impact to be set into motion. It is this difference which makes dialectics such an appealing approach, since causation does not demand a ‘first cause’ and no metaphysic instance needs to be assumed. Motion is simply a basic principle of the matter itself.

**Dialectical Materialism in Holzkamp’s Critical Psychology.** Holzkamp assumes a dialectical materialism, which inherently rejects the mechanistic concept of materialism. In materialist dialectics, a phenomenon is understood through its history, by understanding how it has historically developed through the dissolution of contradictions into new forms of unity. Therefore, the way to comprehend the (human) psyche is to understand its development, i.e. the contradictions resolved during development and the new unities arising from this. Consequently, Holzkamp approaches different psychic categories, such as motivation, emotion or attention, revealing them as units related to one another and to the human psyche as a whole (Holzkamp, 1985; Tolman, 1994, p. 71). Holzkamp incorporates dialectical principles in his approach as he states that:

“It is essential to reconstruct the categories that are being investigated in psychology — the parts that form its totality. It is necessary to consider that many of these categories have a short scientific history but a long history as such. Therefore, the reconstruction has to begin with the emergence of the subject in natural history”.

The development of the human psyche and its material basis begins with the phylogenetic evolution, which Holzkamp conceptualises as a process of dialectical motion. Importantly, in his accounts on phylogenetic emergence, Holzkamp refers to the dialectical principle of the transition from quantitative to qualitative development (1985, p. 78). For neuropsychology, unveiling the dynamics of the transformation of quantitative to qualitative development equals the understanding of the interrelations between the units of functional psychic organization.

**Dialectical Materialism in Vygotsky’s Approach.** Vygotsky asserts that his “dialectic of psychology is at the same time the dialectic of man as the object of psychology, just as the dialectic of the natural sciences is at the same time the dialectic of nature” (Vygotsky, 1997b, p. 256). The principles of dialectics, on which Vygotsky bases his methodology, align with those that Holzkamp applies to his critical psychology. Following the principles of dialectics, rather than excluding contradictions as negative to being, they are understood as the motor of development. Instead of attributing every effect to external causes, things are understood according to their own movement. In the things themselves poles of contradiction collide, leading to their transformation. Contradictions dissolve into unity by different means, including subordination of one pole under another, dominion of one pole over another (Vygotsky, 1997b, p. 239), merging together of their poles, or conversion of one pole into the other (Vygotsky, 1999c, p. 43). Within the new unity, the poles of contradiction are not lost, but they continue to exist in a new relation to the whole and towards each other (cf. Kozulin, 1986, p. XVII). Consequently, for Vygotsky, explaining psychic processes as the result of the dissolution of contradictions into new unities within their development, equals the question of the interrelation of their poles (Vygotsky, 1999b, p. 58).

The implications of this approach are various. Dialectical materialism assumes that phenomena emerge due to the dissolution of contradictions into new unities. Vygotsky’s reliance on this principle is well documented in his works (e.g. Vygotsky, 1999a, p. 37). Viewing phenomena as dialectical unities implies to accept their incorporation of historical contradictions as units, and not to solely rely on their (atomic) elements. In this way, development becomes an indispensable principle to explain phenomena. The principle, of contradictions as the motor of development, can be seen in various accounts of Vygotsky’s work (cf. del Rio, 2002). An important implication for the reconstruction of development is that not only the relations between units may change within the course of development (cf. Kozulin, 1986, p. XXXI), but also the dynamics of the development itself can be altered within the process. The emergence of psychic functions is, according to Vygotsky’s approach, the result of “three main lines in the development of behavior—evolutionary, historical, and ontogenetic” (Wertsch, 1993, p. X), each following its proper principles (Vygotsky & Luria, 1993, p. 37). Holzkamp acknowledges this peculiarity of psycho-genesis as well, writing that psychic development begins as a natural, phylogenetic process, governed by the laws of natural selection transforming into a process determined by the principles of socio-historical development (after a transitional period in which the new social principles themselves become naturally manifested; Holzkamp, 1985, p. 159f.). The phylogenetic principles of development impact human psychic functions therefore due to the formation of their fundamental units. However, with the establishment of socio-historical mechanisms of development, the natural psyche is superseded within a new psychic whole, which does not directly depend on natural selection (Vygotsky & Luria,^1993^, p. 78). Naturally, the dialectical approach implies the relevance of the interrelations of any unit of a complex whole, and thus highlights the importance of the basic units for the development of the higher ones. Even though phylogenetic principles do no longer dominate the current development of human psychic processes, their impact on the formation of the basic units of the psyche needs to be unveiled if one aspires to disclose its complex dialectical structure (cf. Nigrini & Esteban-Guitart, 2023). In their accounts on phylogenetic emergence, Vygotsky and Luria apply the dialectical principle of the transformation of quantitative into qualitative development (cf. Vygotsky & Luria, 1993, p. 63; Vygotsky, 1999a, p. 3). Similarly to Holzkamp, Vygotsky and Luria apply this principle assuming that qualitatively new phenomena arise from quantitative changes, which continuously evolve from the inner contradiction of a thing i.e., from its own movement. Importantly, a dialectical understanding of this principle does not refer to a quantitative to qualitative shift in characters of elements or atoms of a thing, but rather to the contradiction in the centre of the development of the phenomenon.

“Contradictions [may be] ‘slumbering’, ‘growing’, ‘intensifying’, ‘increasing’, ‘sharpening’ [since] the contradictions are constantly effective, but not always at the peak of their development […]. Their effects are different according to the degree of their development; in their lower grades they show themselves as driving forces primarily in development, in their higher grades primarily as those of destruction” (Conze, 2016, p. 291).

The principle that quantity transforms into quality not only corresponds to phylogenetic development of mental functions and their (nervous) organisation but also to their ontogenetic development. This implies the idea of the neoformation of mental functions within the course of ontogenetic development. In Vygotsky’s approach, human psychic processes and their organisation are essentially dynamic: they are to be found within a prolonged process of rising in contradictions, forming new unities and being reorganised as parts of a new complex whole (cf. Del Río & Álvarez, 2011). Reflecting on this dynamic character of psychic functions, Luria states that “functions may, at different stages of development, be accomplished by different parts of the cortex” and that the “relation of separate cortical zones is changed in the process of development” (1965, p. 392). This importantly implies that in neuropsychology, the dynamic character of psychic functions must be considered when investigating their neural substrate.

### Holzkamp’s and Vygotsky’s Position towards the Second Assumption of (Neuro-) Psychological Mainstream

Mainstream neuropsychology assumes and applies formal logic, which implies mechanistic materialism and therefore excludes every notion of becoming. It is a logic of the being, where contradictions are understood as equal to not-being. Since mechanistic materialism attributes the cause of every change to something external, liberating the things from anything that is not in rest, it is a consistent ontological category for formal logic. Holzkamp’s and Vygotsky’s approach, in contrast, being based on dialectical materialism and thus on the understanding of contradictions as the real sources of development (Holz, 2005, p. VIII), contains features opposing the premises of formal logic. Jäger and Leiser remark that lacking a material basis of its categorical terms, in the psychological mainstream, formal logic becomes the factual determinant of the scientific form of psychology (1979, p. 32). As an example, they refer to Holzkamp’s idea of the so called ‘norm-test subject’, a concept (implicitly) used in mainstream psychology to refer to a person without individual characteristics and without history (cf. Tolman, 1994, p. 129). According to Holzkamp the concept describes

“an imaginary person that completely complies with the agreements of the experiment, [and] solely does exactly what the experimenter ‘inserted’ into her. [T]he purpose of experimental planning and of data analysis is to be understood as completed if one is able to extinct or isolate everything that distinguishes a real person from the imaginary, ideal, norm-test subject” (Holzkamp, 1985, p. 52).

The norm-test subject is thought as passive, and in rest. Formal logic applies to it because it is not subject to change. Regarding the norm-test subject, statements about being, as in remaining, are possible without any doubt. This concept perfectly applies the principles mechanistic materialism, since change here is only a product of external factors. This concept is well suited for mainstream psychology, but it does not resemble anything close to a real person. Vygotsky as well criticises the formal logic methodology of psychologists, who like

“Gutmann […] take[…] the formal-logical position. He [Gutmann] considers the relation of speech and action as a thing and not as a process; he takes it statically and not dynamically, not in movement; he considers it as eternal and unchangeable, while it is historical and assumes a different concrete expression at each stage of development” (Vygotsky, 1999d, p. 66).

Vygotsky argues that theories of the psychological mainstream “[w]hether inclining toward pure naturalism or extreme idealism, […] have one trait in common —their antihistorical bias” (Vygotsky, 1986, p. 225), which can easily be traced back to their formal logic basis. Vygotsky’s attitude towards the use of formal logic in psychological theorising translates into neuropsychology as the rejection to study higher mental functions which are already matured (Veresov, 2010). Vygotsky’s conception of mental functions is essentially dynamic and prioritizes the genetic method over (solely) analytical means. Holzkamp’s concept of the norm-test subject applies as well to the field of neuropsychology. Agreements between participants and experimenters are equally made here, limiting the range of behaviours shown as response to experimental situations. Thus, the antihistorical bias is being expressed as the negation of a subject’s history. An example of this in neuropsychology is the merging together of individual structural and functional measures of the brain where the result is read as some kind of purified outcome – without individual difference, without history in imaging studies. Formal logic is unable to grasp development, since history cannot be represented adequately within this framework. Change is only considered as a function of an external impact, as the result of a psychic disorder or drug, for instance.

### Position of Holzkamp’s Critical Psychology towards the Third Assumption of (Neuro-) Psychological Mainstream

Within the neuropsychological mainstream, the tendency towards analytic decomposition (atomism) of psychic phenomena regarding the psyche is prevailing. There, the phenomenal whole is held to be the mere product of its parts, which in return are understood as external properties that can be explained based on their own laws, rather than from the perspective of the whole. According to this methodology, psychic phenomena such as consciousness or executive function can be reduced to neurotransmission, chemistry, and genetics. Vygotsky expresses a very clear opposition towards this approach to complex psychic phenomena (Vygotsky, 1986, p. 1). He illustrates his critique by saying that “The first method [analytic decomposition] analyses complex psychological wholes into elements. It may be compared to the chemical analysis of water into hydrogen and oxygen, neither of which possesses the properties of the whole and each of which possesses properties not present in the whole” (Vygotsky, 1986, p. 4). In neuropsychology the analytic/ atomist approach leads to some specific deficiencies: “[a] substantial amount of neuropsychological research […] is dedicated to studying functions of certain brain regions as if they can be isolated from the whole system that underlies a psychological process” (Toomela, 2014, p. 332). In treating the functions of single brain regions as (quasi) independent from the overall psychic functioning, they are de facto treated as being external to the complex whole. Such an approach towards psychic processes is doomed to neglect the interrelations between the parts that constitute the psychic unity. The dialectical approach, in contrast, understands the parts of a whole as integral parts (units) of a unity, rather than something external, so that the functionality of the units is only to be grasped from the perspective of the whole, which needs to be understood through its history (Holzkamp, 1985, p. 49). Importantly, the “properties of the components [units] change when they enter into a higher level [new unity]” (Toomela, 2014, p. 318). In terms of the development of the nervous system, this means that “the evolution cannot solely be explained by the chemical organisation of the organisms, but must also consider the proper active, functional, biological organisation of the different stages within the evolutionary development” (Del Río & Álvarez, 2011, p. 603). Holzkamp applies the dialectical principle of analysis into units within his psycho-phylogenetic analysis, where he describes the subordination of biological developmental principles to social ones (cf. Tolman, 1994, p. 86-88). He assumes that within the process of hominization, social organisation for individual survival emerges as an evolutionary functional adaptation, which is to become dominant for the subsequent developmental process as such (cf. Holzkamp, 1985, p. 189). Holzkamp and his colleagues, like Volker Schurig, describe a transitional phase in hominization in which natural and cultural poles of the developmental contradiction merge together as ‘Animal-Human-Transitional-Zone’ [*Tier-Mensch-Übergangsfeld*]. Within this period of transition, social forms of behaviour emerge, which are eventually to assume dominance over natural ones, while at the same time natural selection and evolutionary mechanisms continue to shape the development of human psychic functions (Schurig, 1976, p. 10). Holzkamp describes that in this period the social organisation of behaviour itself becomes an evolutionary advantage, thus leading to the manifestation of a ‘social nature’ through means of natural development (Holzkamp, 1985, p. 180 f.; cf. González Rey, 2019, p. 84).Also, Vygotsky acknowledges the shift from phylogenetic developmental principles towards socio-historical ones: “the primitive or natural stage is not replaced by later cultural stages, rather the latter was superimposed […] on top of the former, changing, restructuring, and adapting these natural processes” (Knox, 1993, p. 10f.), and thus expressing the superseding of natural principles of development within cultural ones (cf. Vygotsky, 1999c, p. 43).

### Position of Holzkamp’s and Vygotsky’s Approach towards the Fourth Assumption of (Neuro-)Psychological Mainstream

Mainstream neuropsychology commonly follows a reductionalist/naturalist logic, which leads it to identify psychic processes and phenomena with biological/chemical ones at the level of neurotransmission or genetics. The idea of the brain as sole carrier of human psychic processes is opposed to a dialectic conception of psychic organisation (Conze, 2016, p. 109). Dialectic materialism does not argue that psychic processes were not reflected within the biological constitution of humans (e.g. within the nervous system), but it draws some very different conclusions compared to mechanistic neuropsychology. Rather than understanding the outside world of an organism as external to it, dialectics suppose a unity of environment and individual. Said unity does not need to lie completely within the organism. This is reflected in the fact that the practical activity of an organism is the criterion on which its functionality in terms of survival is based (Conze, 2016, p. 109). Practical activity is a unity of the organism-pole and the environmental pole, therefore psychic processes (which are adaptive activities) do not need to lie completely inside the organism. Vygotsky expresses this thought as well, since he, according to del Rio and Álvarez, “gives his model two firmer, material anchors: one in the external environment (the extra-cerebral connections) and another inside the organism (the change in intra-cerebral connections)” (Del Río & Álvarez, 2017, p. 71; cf. Vygotsky, 1997a). Psychic activity is therefore the result of an organism-pole and environmental pole resolving into unity.

During natural development, the synthesis of the dissolving developmental contradiction has predominantly been reflected in the subsequent organism pole. However, with the emergence of the social mode of survival, human influence on the environment intensified. The socially mediated form of survival is in the beginning an evolutionary adaption, later however it becomes the primary principle of development (Holzkamp, 1985, p. 178; Tolman, 1994, p. 86-88). It refers to the principle that, rather than individual characteristics, the social nexus decides over survival of group members. This indirectness between individual and survival means that individuals gain a certain distance from biological necessary activities. Individuals can engage activities that standing alone would not be sufficient for their survival (they gain a gnostic distance [*gnostische Distanz*]; Holzkamp, 1985, p. 422). Due to this distance between immediate activity and the general nexus of survival, new forms of activity can emerge. Following Vygotsky’s thought del Rio and Álvarez explain that

“[i]t is quite remarkable that man possesses exceptional freedom to intentionally take any action, even foolish actions. That freedom is a characteristic of civilized man, to a lesser degree it is even one of a child and probably of primitive man, and distinguishes man from his closest animal relatives” (Del Río & Álvarez, 2017, p. 79).

Given that individuals in a socially mediated way of life are not required to do what is immanently necessary, actions can be performed in anticipation of future needs. This allows the emergence of tool production. Thereby, tools are crafted not for immediate purposes but for potential future use —a concept Holzkamp terms the ‘Purpose-means reversal’ [*Zweck-Mittel Umkehr*] (Holzkamp, 1985, p. 173; cf. Tolman, 1994, p. 91). The specific human relation between the manipulation of the environment and the adaptation to the (manipulated) characters of the environment can be described with the two terms of objectification [*Vergegenständlichung*] and appropriation [*Aneignung*]. Holzkamp describes how the appropriation of the meaning of objectifications (or more generally of social relations) as human-made things for the purpose of reproducing a certain social context, becomes a human necessity. Vygotsky similarly underscores the primacy of social factors for the psychic activity: “Human behaviour is the product of development of a broader system than just the system or a person’s individual functions, specifically, systems of social connections and relations, of collective forms of behaviour and social cooperation” (Vygotsky, 1999c, p. 41). Although not every individual needs to appropriate the whole spectrum of social meanings, a society needs to sufficiently appropriate them, on average. Holzkamp does not specify how the appropriation of social meanings is reflected within the functional psychic organisation. However, he posits that such appropriations fundamentally shape the very basics of the human psyche, like perception (Holzkamp et al., 2006, p. 151). Further, Holzkamp suggests that the reflection of these appropriations within psychic functions is not emergent by means of phylogenetic adaption. Consequently, it follows that human psychic functions need to be reflected within a flexible psychic organisation, to adapt to rapid social changes. Schurig (1976, p. 75) further proposes the development of behavioural plasticity during hominoid evolution as a precursor to socially mediated survival in humans. Moreover, he describes how the organisational characteristics of human psyche (consciousness), like the integration of information within the forebrain, appear already in human predecessors (Schurig, 1976, p. 136). This observation supports the assumption of material dialectics that quantitative change turns into qualitative change. Importantly, social relations/ social meanings are not ‘stored’ within the inner psychic organisation, but are rather represented within objectifications of social relations (Schurig, 1976, p. 317). Since social meanings change rapidly depending on many individual factors, such as individual position within the context of social reproduction (Nigrini & Esteban-Guitart, 2023, p. 14), their individual reflection and underlying physiological substrate must follow dynamic principles. Schurig (1976, p. 28) suspects that the cerebral cortex may play a role within the emergence of consciousness and dynamically reflects the appropriation of social relations.

Vygotsky expresses similar ideas regarding the organisation of psychic activity in his eco-functional approach. He states that social relations cannot be represented by means of the natural development of psychic functions. Instead, they have to become (re-)represented. The (re-)representation reflects the social meanings of things instead of their natural (physical/biological) properties. Social meanings, inherent in the external stimuli/factors must be (re-)represented (Del Río & Álvarez, 2017, p. 78). The integration of external factors into psychic processes proceeds from a position in which they form part of a so called ‘extra-cerebral organisation’. Here, additionally to intra-cerebral (synaptic) connections, ‘extra-cerebral connections’ exist between the internal psychic organisation and external factors, which “extend […] the internal brain and reconnect [it] in a new way” (del Rio, 2002, p. 244). The extra-cerebral organisation of psychic functions serves the realization of concrete objectives as Vygotsky mentions that “[a]uxiliary stimuli [speech in this case] that fulfil a specific function of organisation of behaviour are nothing other than the symbolic signs that we have been considering here” (Vygotsky, 1999a, p. 16 f.). However, apart from the organisation of activities through internal organisation and external connections, the external connections contribute to establishing new internal connections, so that “the role of external factors (stimulus-mediators, symbols) in establishing functional connections between various brain systems is, in principle, universal” (Kotik-Friedgut & Ardila, 2014, p. 378). The formation of extra-cerebral connections re-organises the internal relations of the brain. The role of external factors in interconnecting the internal psychic organisation becomes obsolete when new, direct internal connections are established. Vygotsky refers to this process as internalisation (Kozulin, 1986, p. XXVI). The external factors which form part of an extra-cerebral organisation are at the same time social relations (signs), which transform the (inner) psychic organisation and also constitute social meanings (i.e., have a purpose within the context of social reproduction). Thus, the internalisation of extra-cerebral connections does not dismiss the role of the sign as external factor, because the internal psychic organisation and the external factor (sign) form a new unity of extra-cerebral relations which supersedes its historical predecessor. This new unity is also expressed in a new attitude towards the sign in terms of activity: the development of higher mental functions (HMF) through the formation and internalisation of extra-cerebral connections follows the logic of dialectical rising in contradictions. Importantly, “[p]rinciple of semiotic mediation and role of culture in Vygotskys theory are not accidental or transient” (Kozulin, 1986, p. LIIIf.). Del Rio and Álvarez say that Vygotsky clearly highlights the notion that “once their constructive role has been fulfilled, the processes of the shared and culturally distributed mind […] perceived so clearly in the developmental process would not disappear” (2017, p. 67). According to Vygotsky’s conception, extra-cerebral organisation is an essential characteristic of HMF. The dialectical principles in the development of HMF are not restricted to change in synaptic connections in cortical areas, as development needs to reflect how lower units are superseded within the neoformations, and thus which new functionalities lower units obtain within the new functional whole. Subcortical organisation, which is primarily the product of the natural line of development, cannot be ignored. However, there is no controversy about the need to also include the substrate of natural development (i.e., subcortical units) into the analysis. Since it appears to be generally accepted that “the natural line is also a line of development [and t]herefore it should be revealed how brain organisation changes in the course of the development” (Toomela, 2014, p. 336).

## Synthesis

### Dialectical Materialism: Holzkamp’s and Vygotsky’s Approach in Comparison

In the development of psychic functions, the contradictions that determine the emergence of a new unity usually consist of an environmental pole and an organism-pole. Both of them, collide within the frame of psychic activity (not necessarily within the nervous organisation). At first sight, it appears to be a major point of theoretical alienation between Holzkamp’s and Vygotsky’s lines of thought, which pole of this contradiction is to assume the primacy within the development of the psychic. On the one hand, the unrestricted preference for the environmental pole leads to a reductionism of psychic development to external causes, ergo to a form of mechanistic materialism that is in itself contradictory. On the other hand, the sole promotion of the organism-pole leads to an intellectualistic conception that resembles ontological idealism, in which things develop teleologically. In both scenarios, the principles of dialectic materialism are violated.

**Flaws in Dialectical Logic: Overemphasis on the External Pole of Development**. Holzkamp’s critical psychology incorporates many references to the writings of A.N. Leontjew, a former collaborator of Vygotsky, who eventually participated in the foundation of the cultural historical activity theory (CHAT). Holzkamp’s references to Leontjew are mainly present within his functional-historical reconstruction of the phylogenetic development of the psyche and its categorical foundation. Here one can find many references to Leontjew’s (1981) “Problems in the development of mind”. This reliance on Leontjew’s work might suggest that Holzkamp’s critical psychology is susceptible to the same flaws as CHAT, which has been criticised for overemphasizing the environmental pole of the contradiction in the development of psychic processes and thus reducing psychic activity to its relation towards a certain environment.

In this context, CHAT is criticised for failing to consider the “possibility that mental structures underlying external task performance may be different” (Toomela, 2014). The overemphasis of CHAT on the environmental pole of development is reflected in the neglection of differences in the organism-pole, as behaviours are considered equal solely on the base of their external outcome. Activity as a unity of organism-pole and environmental pole can be reached in different ways, though external similarity does not necessarily imply that two unities are the same, since their composition out of different units may vary significantly. Holzkamp however does not follow this reductionist approach, and rather accepts the notion of activity as unity of organism-pole and environmental pole. Neglecting the organism-pole of development leads CHAT to a reduction of (individual) activity to social circumstances (cf. González Rey, 2019, p. 82). Vygotsky criticises this tendency, which has resulted in the “derivation of a psychology solely on the basis of Kapital” (Kozulin, 1986, p. XXIII). CHAT seems to commit this error by using “the categories of appropriation and objectivation [sic] which apply to the socio-historical subject rather than […] to the psychological individual” (Kozulin, 1986, p. In fact, Holzkamp refers extensively to these categories. As previously discussed, the incautious use of these categories might lead to a reduction of individual behaviour to societal circumstances, and thus to the environmental pole. Both objectification and appropriation describe societal necessities within the context of the reproduction of a certain social nexus, which forms the very basis of human survival, since individuals’ activities only by mediation of the socio-productive context contribute to their own survival. Holzkamp acknowledges the social mediation of survival, but does not commit the reductionist error (cf. González Rey, 2019, p. 84). He assumes that the social mediation of behaviour also provides individuals with a gnostic distance (Holzkamp, 1985, p. 422) due to which individuals are not determined by the social context. Rather, individuals are located in a relation of possibility [*Möglichkeitsbeziehung*] towards society and activities implied in social reproduction, such as objectification and appropriation (Holzkamp, 1985, p. 321 f.; Tolman, 1994, p. 33). The specific non-deterministic relationship between individual and society allows the consideration of the organism-pole of the developmental contradiction, which in a neuropsychological approach corresponds to the consideration of certain characteristics of the internal functional organisation of psyche. In Holzkamp’s categorical analysis, this notion is reflected in the term of ‘subjectivity’, which he applies to describe various phenomena belonging to the sphere of the organism-pole of development, such as ‘subjective meaning structure’ (cf. Holzkamp, 1985, p. 357). Having established that Holzkamp does not neglect the organism-pole of development and thus overcomes the flaws of CHAT, one may conclude that, unlike CHAT, critical psychology cannot be criticized for being adevelopmental through neglecting the organism pole of the developmental contradiction (cf. Toomela, 2000), which would lead to an approximation toward mechanistic thought.

**Flaws in Dialectical Logic: Overemphasis on the Internal Pole of Development**. According to Vygotsky’s law of double formation “[e]ach function in the child’s development appears twice; (…) first between people (interpsychological), and then inside the child (intrapsychological)” (Kozulin, 1986, p. XXVI). This has been interpreted as the contradiction of organism-pole and environmental pole, which dissolves into the unity of (psychic) activity, successively becoming a property of the internal organisation only within the process of psychic development (cf. Kozulin, 1986, p. XXXVf.). This understanding implies the problematic issue that the environmental pole of development at some point ceases to contribute to the psychic development and functioning, making the social relations, which are implied in the psychic organisation on the side of the environmental pole, mere means of the formation of (internal) cognitive abilities. This position carries very problematic notions for the comprehension of the developmental dynamics of psychic functions. Luria writes that at some point “[d]evelopment finally arrives at a stage when these external auxiliary devices are abandoned and rendered useless” (Luria, 1993, p. 207). This idea implies restricting the role of the environmental pole to a discrete period in the development of HMF and neglects its significance for both the further parttaking in the development and the organisation of psychic activity. The alleged tendency to overemphasize the organism pole of activity in some of his work is suggested to depend on Vygotsky’s misconception about the qualitative transformation from natural to social developmental laws in phylogeny (cf. Wertsch, 1993, p. XI). However, Vygotsky’s objective is clearly to approach this transformation with a dialectical materialist explanation. Thus, the new social developmental principle must emerge on the basis of previous quantitative developments within the natural line of developmental principles. Vygotsky accounts for this phase of development by referring to differentiation of tradition-building, which for Vygotsky is the quantitative basis for the emergence of social mediation in humans. While both humans and apes engage in tradition-building, humans establish a distinct nexus of social cooperative labour, i.e., a nexus of socially mediated survival (Vygotsky & Luria, 1993, p. 17). However, it would be premature to assume that this nexus emerges solely based on primate-like tradition learning. Assuming tradition-building as the only basis of social mediation and social relations, like cooperative behaviours, would imply them to be emerging in principle accidentally and becoming part of the repertoire of human activity only through canonisation. Thus, following the logic of tradition-building, social relations would be considered to emerge more or less coincidentally, even though they are the means of transforming natural mental functions into HMF. This conception implies the absence of an inherent relation between them, so that social relations/signs would appear as an external force in the development of HMF, as in a mechanistic notion of development. Without assuming an internal connection between social relations and natural functions, the former are to be excluded from the equation once their role in imposing a developmental movement is fulfilled: being external to the motion, they would not become reflected as units in the newly emergent unity and would not maintain a dynamic relation with it. Holzkamp disagrees with this perspective, asserting that social mediation is inherently related to natural development. He supposes the existence of a period in which both socio-historical and natural laws of development are in vigour, shaping the natural constitution of humans through the social mode of survival. This leads him to the conception of the “social nature” (Holzkamp, 1985, p. 180 f.; cf. González Rey, 2019, p. 84), a reflection of the socially mediated way of living within the natural properties. By assuming a natural tendency toward social mediation, tradition-building does not remain the sole basis of the formation of society. The social character of human survival is not established in ontogenesis but is innate to every human being from the moment of its birth, and the social nature is the connecting piece between natural mental functions and HMF. It seems that Vygotsky coincides with Holzkamp in the assumption of a social nature (e.g. Vygotsky, 1999a, p. 20). Further, he revisited his initial approach to the development of HMF, as described by the law of double formation, formulating a more explicit dialectical understanding, which highlights the dynamic character of HMF. His understanding of social mediation and the role of social relations clearly excludes the sole reliance on tradition-building. Thereby, social relations as part of the environmental pole are understood in their continued contribution to the unity of psychic action. “Vygotsky already intuits the external corticality as systemic and permanent” (Del Río & Álvarez, 2017, p. 67).

## Discussion: New Horizons for Dialectical Methodology in Neuropsychology

Overall, dialectical materialism appears to provide a more appropriate methodological framework for neuropsychology than currently predominating mechanistic materialism. The main issues with mechanistic materialism include open causal chains, which lead to the necessary assumption of metaphysical ‘first causes’, difficulties to grasp change and development and the understanding of the functional relations between a phenomenal whole and its constituent parts. In contrast, the dialectical approach addresses these problems effectively. Instead of relying on metaphysical ‘first causes’, dialectical materialism assumes own-movement, where things are constantly in development without the need for external causes. Development itself is grasped as the resolution of contradictions, with poles of contradiction being superseded within new unities that establish new interrelations. Understanding a thing and its elements in dialectics entails grasping its history of rising in contradictions.

Both Vygotsky and Holzkamp have developed comprehensive and impeccable dialectical approaches, which have significant implications for neuropsychology. Their impact has been and continues to be considerable. Nonetheless, there are aspects of neuropsychology yet to be subjected to this dialectical approach. Holzkamp’s approach, influenced by CHAT, may lack the depth of structural understanding that Vygotsky has developed, possibly due to a stronger focus on the environmental pole at the expenses of the organism-pole and its units. On the other hand, Vygotsky’s approach has not been fully extended towards the study of subcortical units, which are closely related to phylogenetic development. This lack of attention may stem from ambiguities in Vygotsky’s early accounts on the role of social relations and the concept of social nature. Subsequent readings may have emphasized cultural development over natural development, leading to the neglect of subcortical units. Consequently, the prospect of expanding the dialectical approach in neuropsychology to include the structural study of phylogenetic development and subcortical units poses a significant challenge for the future. Addressing this challenge will require integrating insights from both Vygotsky’s and Holzkamp’s approaches to create a more comprehensive framework for understanding the development of the human psyche from both cultural and natural perspectives.

## Data Availability

No datasets were generated or analysed during the current study.
